# Ancient Host–Pathogen Associations Maintained by Specificity of Chemotaxis and Antibiosis

**DOI:** 10.1371/journal.pbio.0040235

**Published:** 2006-07-11

**Authors:** Nicole M Gerardo, Sarah R Jacobs, Cameron R Currie, Ulrich G Mueller

**Affiliations:** **1**Section of Integrative Biology, University of Texas at Austin, Austin, Texas, United States of America; **2**Smithsonian Tropical Research Institute, Balboa, Republic of Panama; **3**Department of Ecology and Evolutionary Biology, University of Arizona, Tucson, Arizona, United States of America; **4**Department of Pharmacology and Cancer Biology, Duke University, Durham, North Carolina, United States of America; **5**Department of Bacteriology, University of Wisconsin at Madison, Madison, Wisconsin, United States of America; University of LausanneSwitzerland

## Abstract

Switching by parasites to novel hosts has profound effects on ecological and evolutionary disease dynamics. Switching requires that parasites are able to establish contact with novel hosts and to overcome host defenses. For most host–parasite associations, it is unclear as to what specific mechanisms prevent infection of novel hosts. Here, we show that parasitic fungal species in the genus *Escovopsis,* which attack and consume the fungi cultivated by fungus-growing ants, are attracted to their hosts via chemotaxis. This response is host-specific: Escovopsis spp. grow towards their natural host cultivars more rapidly than towards other closely related fungi. Moreover, the cultivated fungi secrete compounds that can suppress *Escovopsis* growth. These antibiotic defenses are likewise specific: in most interactions, cultivars can inhibit growth of Escovopsis spp. not known to infect them in nature but cannot inhibit isolates of their naturally infecting pathogens *.* Cases in which cultivars are susceptible to novel *Escovopsis* are limited to a narrow set of host–parasite strain combinations. Targeted chemotactic and antibiotic responses therefore explain why *Escovopsis* pathogens do not readily switch to novel hosts, consequently constraining long-term dynamics of host–parasite coevolution within this ancient association.

## Abstract 2

Ant-cultivated fungus gardens are attacked by a pathogenic fungus called *Escovopsis.* Here the authors demonstrate the exquisite specialization of different *Escovopsis* pathogens for particular fungal cultivar strains.

## Introduction

Recent public concern over possible acquisition of virulent diseases from nonhuman hosts highlights the need for understanding the ecological and coevolutionary maintenance of pathogen host fidelity [ 1– 3]. Under what conditions are pathogens host-specific, and under what conditions do pathogens switch to novel hosts? Switching hosts requires that a parasite is able to establish and maintain infection on a new host. To achieve this, a parasite must contact a potential host, overcome host defenses, use that host as a resource, and maintain that association long enough to replicate and spread to new individuals. Over time, as hosts evolve defenses against parasites to avoid or attenuate infection, parasites counter-adapt to circumvent these specific defenses. This arms-race process of coevolution can lead to the specialization of parasites on their current hosts such that they are incapable of switching to novel hosts [ 4].

Taxonomically diverse parasites (e.g., insects, bacteria, fungi, birds, and nematodes) exploit host-derived cues to find susceptible hosts [ 5– 8]. Cues may be visual or behavioral [ 9] but are most frequently chemical [ 6]. These cues are likely to vary between, and possibly within, host species. Only a limited number of studies have compared pathogen recognition of cues from a range of potential hosts to determine whether attraction is specific to host over non-host cues [ 10].

For successful infection, a pathogen must not only locate a host but must also overcome host defenses. Similar to attractants, defenses may be highly specific. Host defenses include production of pathogen-inhibiting antibiotics [ 11], activation of immune responses, and physical removal of parasites [ 12]. Some of these are general responses to a wide range of parasites, while others, such as human adaptive immune responses, may be specific to a particular pathogen strain.

Understanding the extent to which host and parasite traits restrict host use requires systems in which genetically diverse hosts and parasites can be confronted in experimental combinations and in which the interactions can be observed easily. The ancient, coevolving fungus-growing ant–microbe symbiosis is one such system where host–pathogen interactions are tractable. Fungus-growing ants have evolved over a period of at least 50 million years along with the fungi that they cultivate as their primary food source [ 13– 15]. With few exceptions, each of the approximately 210 described ant species grows only a narrow range of genetically similar fungal cultivar types. This specialized mutualism is exploited by *Escovopsis,* a diverse genus of pathogenic fungi that attack and consume the ants' fungal cultivars [ 16, 17]. Morphologically and genetically distinct *Escovopsis* types each infect a narrow range of cultivar hosts, suggesting that ancient coevolutionary processes define the association of the ants, their cultivars, and *Escovopsis* [ 18]. The genetic diversity of the hosts and parasites in this system, coupled with the known specificity of pathogens, makes it an exceptional system in which to study both ecological and evolutionary host–pathogen dynamics.

**Figure 1:**
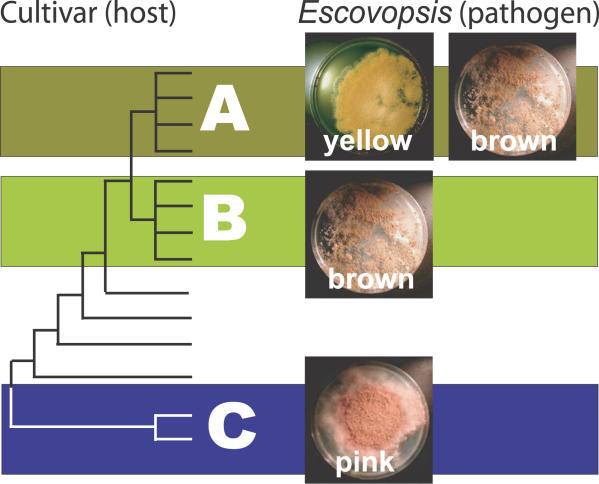
Schematic Diagram of the *Escovopsis*–Cultivar System Fungus-growing ants in the genus *Apterostigma* cultivate fungi in three distinct groups. Cultivars in clade C (referred to in the text as cultivar C) are lepiotaceous fungi distantly related to the pterulaceous cultivars in clades A and B. Free-living, nonsymbiotic fungi fall between clade C and the other cultivars. Each cultivar clade is attacked by specific *Escovopsis* types, here identified according to spore color. Note that the same brown *Escovopsis* attacks both cultivars A and B. Schematic cultivar phylogeny based on [ 33].

Through microbial bioassays confronting cultivar hosts and *Escovopsis* parasites, we examine first whether *Escovopsis* is able to recognize and grow towards cultivars, and second, whether cultivars can defend against infection. Coupling genetic and experimental analyses utilizing morphologically diverse *Escovopsis* and cultivars isolated from colonies of *Apterostigma* ants ( Figure 1), we further investigate whether host and pathogen responses depend upon the combination of host and parasite genotypes involved. These investigations shed light on how genotype-specific adaptations may prevent host switching and thus maintain long-term host–parasite fidelity within this ancient microbial interaction.

## Results

**Figure 2:**
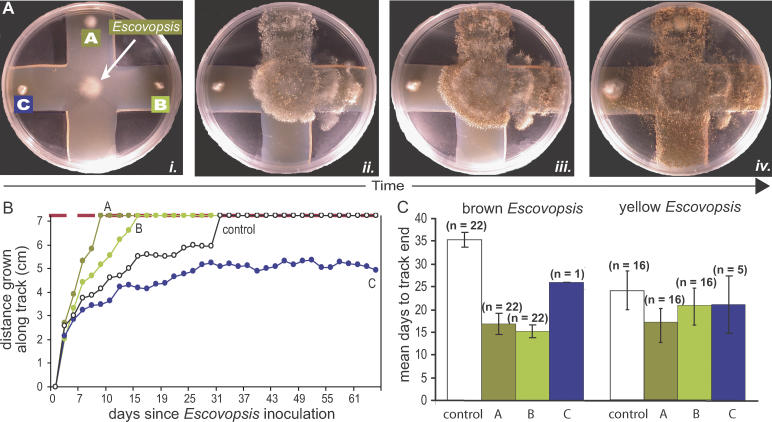
Fungal-Choice Bioassays (A) Isolates of cultivars A, B, and C are placed at the end of each of three tracks, and one track remains blank as a control. Brown *Escovopsis* begins to grow concentrically (i), but over time overgrows cultivar A (ii), then cultivar B (iii), then the end of the control track (iv) *.* After several months, the parasite has still not overcome the zone of inhibition surrounding cultivar C (iv). (B) Progression of *Escovopsis* growth over time on the plate shown in (A); the red, dashed line indicates the track end, at which the pathogen overgrows the cultivar. (C) Average number of days to reach each cultivar type in fungal-choice bioassays. For cultivar C, results are included only for trials in which *Escovopsis* was able to overgrow cultivar C and reach the end of the track.

We discovered that, on standard media, *Escovopsis* is attracted to chemical signatures produced by host cultivars, growing rapidly towards these cues. In “fungal choice” trials ( Figure 2A and 2B), most isolates of yellow-spored and brown-spored *Escovopsis* ( Figure 1) were attracted to isolates of their natural hosts and closely related cultivars (cultivars A and B, Figure 1), arriving more rapidly at the ends of these tracks than at the ends of the control tracks ( *p* < 0.01 in a least-squares mean comparison of both clade-A cultivar [cultivar A] versus control and clade-B cultivar [cultivar B] versus control for both brown and yellow *Escovopsis;* Figure 2C). More specifically, yellow *Escovopsis,* which uses only cultivar A as a host in nature, was more rapidly attracted to cultivar-A isolates than to isolates of the closely related cultivar B ( *p* = 0.03; Figure 2C), whereas brown *Escovopsis,* which uses both cultivars A and B as hosts, did not show preferential attraction between these two cultivar types ( *p* = 0.86; Figure 2C).

In most of these fungal-choice bioassays, yellow and brown *Escovopsis* isolates were inhibited by clade-C cultivar (cultivar C), which is distantly related to their natural hosts. In 11 out of 16 yellow *Escovopsis* choice bioassays and in 21 out of 22 brown *Escovopsis* choice bioassays, even after several months, a zone of inhibition surrounded cultivar-C isolates, and the pathogens could not establish infection ( Figure 2A, iv). In the six trials in which yellow and brown *Escovopsis* did overgrow the cultivar-C isolate, there was no statistical support for more rapid growth to the cultivar-C isolate than to the end of the control track (Wilcoxon rank sum test: control versus cultivar C, *V* = 12, *p* = 0.88).

**Figure 3:**
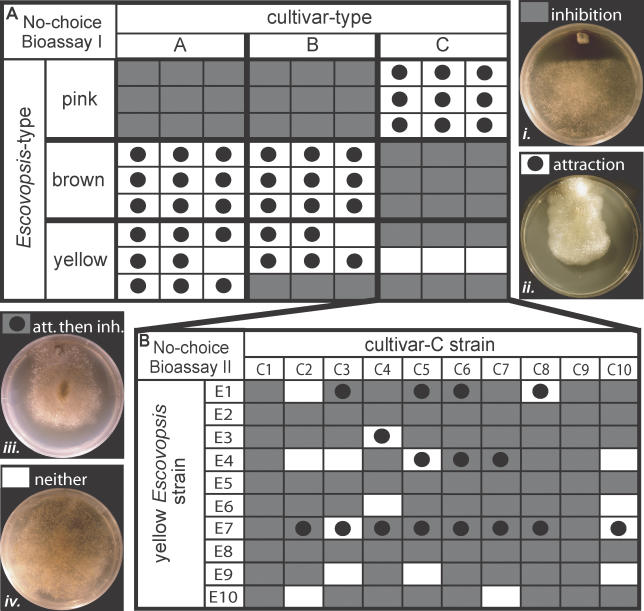
No-Choice Bioassays Each cell represents the outcome of the interaction between one cultivar and one *Escovopsis* isolate. Gray indicates inhibition (i), a dot indicates attraction and subsequent infection (ii), gray with a dot indicates attraction followed by inhibition (iii, note that the cultivar is not overgrown), and white indicates neither attraction nor inhibition (iv). (A) No-choice bioassay I. Interaction between three isolates of cultivars A, B, and C and three isolates of each of the *Escovopsis* morphotypes. (B) No-choice bioassay II. Interaction of ten yellow *Escovopsis* isolates confronted with ten cultivar-C isolates. These 100 bioassays are a more exhaustive dataset corresponding to the nine yellow *Escovopsis*–cultivar C trials in no-choice bioassay I.

Because fungal-choice bioassays indicated that cultivar C typically inhibits yellow and brown *Escovopsis,* pathogens that do not attack cultivar C in nature, we chose to then ask whether cultivar C would also inhibit pink *Escovopsis,* which does infect it in nature. Fungal-choice bioassays with pink *Escovopsis* were not possible owing to limitations of sample size and the slower growth of this *Escovopsis* as compared to yellow and brown *Escovopsis.* Therefore, to investigate cultivar interactions with pink *Escovopsis,* we conducted “no choice” bioassays interacting a single cultivar and a single pathogen isolate in a smaller arena than used in the choice bioassays. For comparison, we conducted bioassays interacting yellow and brown *Escovopsis* with the same cultivar isolates. Results of no-choice bioassays supported findings from choice bioassays: yellow and brown *Escovopsis* were typically attracted to their natural hosts and closely related cultivars (cultivars A and B) but were inhibited by isolates of cultivar C. Pink *Escovopsis* also typically was attracted to its natural host but inhibited by atypical host cultivars; in six of nine bioassays, pink *Escovopsis* isolates were attracted to isolates of cultivar C, and in 18 of 18 bioassays, the same pink *Escovopsis* isolates were inhibited by isolates of cultivars A and B, which are not natural hosts for pink *Escovopsis* (no-choice bioassay I, Figure 3A).

In the fungal-choice and no-choice bioassays discussed above, interaction outcome varied the most when yellow *Escovopsis* was in the presence of cultivar C. Specifically, in five out of 16 fungal-choice trials ( Figure 2C) and in three out of nine no-choice trials ( Figure 3A), yellow *Escovopsis* isolates were not inhibited by cultivar C. To explore the frequency at which yellow *Escovopsis* can infect its nonnative host, cultivar C, we conducted 100 additional no-choice bioassays challenging strains of yellow *Escovopsis* and cultivar C (no-choice bioassay II). In 15 out of 100 challenges, the yellow *Escovopsis* strain was not inhibited by the cultivar C strain, and in 16 out of these same 100 challenges, yellow *Escovopsis* was attracted to this atypical host ( Figure 3B).

We then asked whether the variation that we observed in interactions between yellow *Escovopsis* isolates and cultivar-C isolates was associated with genetic differences between strains. There was a significant correlation between the genetic distances of cultivar strains in no-choice bioassay II and their inhibition patterns (Mantel test: *r* = 0.43, *p* = 0.04). This indicates that genetically similar cultivar strains were more likely to inhibit the same *Escovopsis* strains than genetically dissimilar strains. Similarly, significant correlation of the genetic distance of *Escovopsis* strains and patterns of inhibition revealed that genetically similar *Escovopsis* strains were more likely to be inhibited by the same cultivar strains than genetically dissimilar *Escovopsis* strains ( *r* = 0.35, *p* < 0.01). Correlations between patterns of attraction and genetic distances were not significant (cultivar *r* = 0.28, *p* = 0.05; Escovopsis r = 0.18, *p* = 0.08).

## Discussion

Although some pathogens can infect diverse hosts, all pathogens have some limit to the range of hosts that they can successfully infect, and most pathogens rarely switch to novel hosts [ 19]. Because a pathogen might increase its reproductive success if it could switch to different hosts when novel hosts are abundant and typical hosts are rare, pathogen specialization on narrow host ranges would appear to be suboptimal. However, specialization can be maintained owing to tradeoffs when pathogen adaptation to one or a few hosts leads to increased fitness on those hosts at a cost of reduced fitness on other hosts [ 4]. As such tradeoff-dependent pathogen evolution is entangled with concurrent evolution of hosts, understanding the exact mechanisms that drive specialization requires the observation of pathogen responses to hosts, as well as host responses to pathogens.

Here, microbial bioassays provide the first evidence that *Escovopsis* can respond to host cues and that cultivars can defend against *Escovopsis.* These responses are specific and may be tied to *Escovopsis*' host specialization. Previous molecular analyses indicate that different *Escovopsis* lineages infect genetically restricted cultivar lineages, suggesting that *Escovopsis* rarely switches between distantly related hosts [ 18, 20]. Follow-up switching experiments in which fungal garden pieces were confronted with typical and novel *Escovopsis* showed that establishment of infection was more likely when a garden piece was confronted experimentally with its naturally associated *Escovopsis* type than with novel *Escovopsis* [ 20]. Our findings suggest that two identified host–parasite adaptations, attraction of parasites to hosts and host defense, may limit infection of novel hosts, helping to maintain *Escovopsis*' host fidelity over both ecological and evolutionary time.

### Parasite Attraction to Host Cues

Patterns of attraction of *Escovopsis* towards cultivars in experimental bioassays are consistent with patterns of host use in nature. In bioassays, all three Escovopsis spp. were more likely to be attracted to isolates of their typical cultivar hosts than to distantly related cultivars. Furthermore, more specialized pathogens had more specialized responses: yellow *Escovopsis,* which attacks only cultivar A in nature, was more rapidly attracted to cultivar A over cultivar B, whereas brown *Escovopsis,* which naturally attacks both of these cultivar types, did not show preferential attraction. Upon arriving at susceptible cultivars, *Escovopsis* quickly overgrew the cultivar isolates, established infection, and presumably degraded the host [ 17].

Paralleling the case of *Escovopsis,* pre-contact attraction to host cues is critical in the life-history of many symbionts, and distantly related parasites use chemotaxis to locate their hosts. Nematodes, for example, are chemotactically attracted to their plant hosts [ 7], and pathogenic bacteria are attracted to substances released by their host fungi [ 21, 22]. Similarly, many pathogenic fungi and fungus-like organisms have zoospores, asexual spores with flagella, which swim towards a wide range of chemicals produced by their plant and fungal hosts [ 10]. Besides *Escovopsis,* few fungi without zoospores are known to exhibit specialized, directed growth towards their hosts [ 23, 24]. One interesting case is arbuscular mycorrhizal fungi, mutualistic associates of plant roots, which are known to be stimulated to grow specifically towards volatile cues produced by host roots [ 25, 26]. Such attraction to host cues is advantageous because it allows both parasites and mutualists to establish contact efficiently with their hosts rather than to depend on random movements to commence association.

### Host Defense against Parasites

In general, host defense is widespread because it benefits any host to defend itself against virulent pathogens. Here, microbial bioassays suggest that cultivars can inhibit many *Escovopsis* isolates, which may prevent the establishment of infection by these pathogens in nature. In most bioassays, cultivar C inhibited yellow and brown, but not pink *Escovopsis,* whereas cultivars A and B often inhibited pink *Escovopsis,* but rarely inhibited yellow or brown *Escovopsis.* Therefore, like *Escovopsis*' attraction to cultivars, patterns of cultivar defense parallel *Escovopsis*–cultivar associations in nature and support the hypothesis that cultivar defenses maintain narrow *Escovopsis* host ranges and prevent host switching.

### Genotype–Genotype Interactions

There were exceptions in which cultivars did not inhibit the pathogens to which they are not naturally associated. In five out of 16 choice bioassays and in 15 out of 100 no-choice bioassays challenging a yellow *Escovopsis* isolate against a cultivar-C isolate, the pathogen was able to overcome host defenses and establish infection. These cases suggest that yellow *Escovopsis* could potentially switch to this novel host.

Correlation analyses between genetic relatedness and outcomes of no-choice bioassays between yellow *Escovopsis* and cultivar C suggest genotype–genotype specificity in this non-host interaction. Genetically similar cultivar C strains are more likely to inhibit the same yellow *Escovopsis* strains than genetically dissimilar cultivars, and genetically similar *Escovopsis* are more likely to be inhibited by the same cultivar strains. Therefore, whether a cultivar strain can successfully suppress an *Escovopsis* strain appears dependent on the genotype combination. Though correlations between genetic relatedness and patterns of attraction were not significant at the *p* < 0.05 level, there may also be a biologically significant association between attraction patterns and genotype.

Two major models describe genetic interactions between hosts and parasites: the matching-alleles model and the gene-for-gene model. Under the matching-alleles model, a parasite's infectivity genotype must exactly match a host's susceptibility genotype in order to establish infection, and all parasites are equally successful when paired with their appropriate host. Under the gene-for-gene model, some parasites are more effective than others, and some hosts are more resistant than others but, owing to associated costs, no genotype spreads to fixation. Biological systems may incorporate both of these dynamics [ 27], and both could lead to correlation between genetic relatedness and infection. Here, no-choice-bioassay interactions between yellow *Escovopsis* and cultivar C are consistent with both models. Sometimes, only exact matches lead to infection, as expected under the matching-alleles model, and there are some yellow *Escovopsis* strains that infect all hosts and some cultivar C strains that are susceptible to all parasites, as expected under the gene-for-gene model. Such genotypic dynamics lead to the maintenance of polymorphism and the fluctuation of both host and parasite genotypes [ 28]. In this case, fluctuations are likely to be driven by interactions between parasites and their typical hosts, but in turn influence interactions between parasites and atypical hosts.

### Conclusions

How may defense and attraction be coupled to maintain *Escovopsis*' host fidelity in nature? Though the mechanism by which *Escovopsis* is transmitted is not known, colonies with the different cultivar types (A, B, and C) can be found only centimeters apart in the field (Gerardo and Currie, unpublished data), increasing the possibility for transmission between hosts. If an *Escovopsis* strain is transmitted to a host with which it is not typically associated, however, the parasite would be able to overcome the cultivar's defenses and establish infection only if the right host and parasite genotypes came into contact. Upon reaching the proximity of a garden with the appropriate cultivar, *Escovopsis* would be able to spread quickly through the garden matrix as it is attracted to portions of the garden with the fungal cultivar. This process could make the establishment of infection more rapid and may prevent the successful suppression of the parasite by the ants, which have behaviors specific to the removal of *Escovopsis* [ 29], and also by filamentous bacteria on the ants, which are known to produce secondary metabolites that inhibit *Escovopsis* growth [ 30, 31].

Over evolutionary time, *Escovopsis*' host fidelity may facilitate cospeciation—the process by which interacting organisms speciate in tandem. Few studies have shown how adaptive evolution may drive cospeciation [ 12, 32]. In the attine ant–microbe symbiosis, both attraction to the host on the part of parasites, as well as host defenses, may limit host switching, reinforcing long-term host–parasite associations and thus facilitating cospeciation. Because these microbial symbionts coevolve with ants and with bacteria on the ants, these adaptations may also indirectly affect the speciation processes of these organisms. Certainly, other adaptations, including ant behaviors, modes of transmission, bacteria-derived antibiotics, and fungal growth properties, are likely to influence the probability of persisting on novel hosts. Future studies should explore the suite of adaptations involved in maintaining specificity of all four symbionts and the pace at which chemical and behavioral adaptations have coevolved over the ancient history of this complex host–pathogen association.

## Materials and Methods

### Collections.

Fungi were cultured from gardens of fungus-growing ants in the genus *Apterostigma* in Panama and Costa Rica following procedures described in [ 20]. Yellow-spored and brown-spored *Escovopsis* isolates ( Figure 1) were from A. dentigerum colonies, which raise cultivar A, while all pink-spored *Escovopsis* isolates were from A. auriculatum colonies, which raise cultivar C ( Figure 1). Besides differences in the color of their conidia (yellow, brown, and pink), the three *Escovopsis* types also have distinct micromorphological differences of their conidiophores (Currie, unpublished data) and fall into three distinct phylogenetic groups (Gerardo et al., unpublished data). No two isolates of the same morphological type were sampled from the same colony.

Cultivar-A isolates were from A. dentigerum colonies; these cultivars fall into the “G2-clade” in [ 14]. Cultivar-B isolates were from *A.* cf. *manni* colonies; these cultivars fall into the “G4-clade” in [ 33]. Cultivar-C isolates were from A. auriculatum colonies; these cultivars fall into the “G3-clade” in [ 14] and, specifically, into “Clade-1” of the G3-clade in [ 34]. Cultivar-A and cultivar-B isolates are in the family Pterulaceae, and cultivar C isolates are in the family Lepiotaceae [ 33].

### Fungal-choice bioassays.

For bioassays with yellow *Escovopsis,* agar in 14-cm petri dishes filled with 50 ml of PDA plus antibiotics (potato dextrose agar with 50 mg/l each of penicillin and streptomycin) was cut out to leave four 4-cm-wide tracks ( Figure 2A). For each plate, each track was randomly assigned to one of four treatments: control (no cultivar), cultivar A, cultivar B, or cultivar C. One of eight cultivar-A isolates, one of eight cultivar-B isolates, and one of four cultivar-C isolates was randomly assigned to each plate. Plates were inoculated with ≈6-mm ^3^ agar pieces covered with mycelium from the appropriate cultivar culture. After 1 wk, plates on which all three cultivar isolates had grown without contamination (27 out of 35) were further inoculated with a ≈6-mm ^3^ agar piece with spores and mycelium of one of 12 randomly assigned *Escovopsis* isolates. Plates were photographed every 1–3 d for 3 mo and were monitored occasionally for several months thereafter. From photographs, we estimated the number of days (#days) that it took *Escovopsis* to reach the end of each track. We excluded data from 11 plates on which *Escovopsis* had not reached the end of the control track within 90 d, because it was unclear whether the cultivars and *Escovopsis* were still viable in these trials.

With data from the remaining 16 plates, we used a random-effects analysis of variance (PROC MIXED, SAS Institute, http://www.sas.com/service/techsup/faq/stat_proc/mixeproc.html) to compare #days (log-transformed) to reach cultivar A, cultivar B, and the end of the control track, treating plate and *Escovopsis* isolate as random effects (in order to account for variation between plates and repetition of parasite isolates) and cultivar type as a fixed effect. We used log-likelihood ratio tests to confirm that there was no effect of random variables, and conducted pairwise, Bonferroni-corrected comparisons of least-squares means of the treatments (A, B, and control).

The fungal-choice bioassays with brown *Escovopsis* were similar, except that we inoculated 26 plates with one of eight cultivar-A, one of two cultivar-B, and one of six cultivar-C isolates and, 1 wk later, with one of ten brown *Escovopsis* isolates. Data from four of these plates were excluded because the date at which *Escovopsis* reached at least one of the tracks could not be estimated accurately from the available photographs; in these cases, photographs were not taken regularly around the time that the parasite reached the track end. Because cultivar C inhibited *Escovopsis* growth on 32 out of the 38 final plates in the dataset, #days to cultivar C was not included in the above analysis of variance. Instead, for trials in which cultivar C was overgrown, combining data from both the brown and yellow *Escovopsis* trials, we used a Wilcoxan rank sum test to compare #days to control versus #days to cultivar C.

### No-choice bioassay I.

For each no-choice bioassay, we placed a single isolate of cultivar near the edge of a 9-cm petri dish with PDA plus antibiotics, as described. After 1 wk, we inoculated the center of each plate with a single *Escovopsis* isolate. Each of nine cultivar isolates (three A, three B, and three C isolates) was interacted with each of the same nine *Escovopsis* isolates (three yellow, three brown, and three pink isolates) for a total of 81 bioassays. Bioassays were monitored for up to 2 mo. Interactions were scored for the presence/absence of inhibition and the presence/absence of attraction.

### No-choice bioassay II.

In a second no-choice experiment, following the same protocol outlined above, ten cultivar-C isolates were interacted with each of ten yellow *Escovopsis* isolates for a total of 100 bioassays. We then used amplified fragment-length polymorphisms (AFLPs) to generate genotype fingerprints of the cultivar strains, following the protocol outlined in [ 20]. To obtain fingerprints of the same level of genetic variability for *Escovopsis,* we sequenced 552 bp of elongation factor 1-alpha, using primers 3F and 5R, following the protocol described in [ 20]. With these data, using PAUP* (version 4b10 [ 35]), we constructed two genetic-distance matrices—a Nei–Li distance matrix for the ten experimental cultivar strains and a maximum-likelihood distance matrix for the ten experimental *Escovopsis* strains ( Figure S1). For maximum-likelihood distances, we used the TrN + Γ + PINVAR model of evolution, where TrN is the Tamura–Nei model of DNA evolution, and PINVAR is the proportion of invariant sites as determined via Modeltest (version 3.06, [ 36]).

Four interaction-distance matrices were constructed ( Figure S1). The first matrix consisted of the inhibition distances between each pair of the ten cultivar C strains, where each inhibition distance ranged from 0 to 1 and increased by 0.1 for each case in which the two cultivar strains had a different inhibition result with the same *Escovopsis* strain (i.e., one cultivar inhibited the *Escovopsis* strain while the other did not). A second matrix consisted of *Escovopsis* inhibition distances; each inhibition distance ranged from 0 to 1 and increased by 0.1 for each case in which the two *Escovopsis* strains had a different inhibition result with the same cultivar strain (i.e., one *Escovopsis* strain was inhibited while the other was not). The third and fourth matrices were comprised of cultivar and *Escovopsis* attraction distances, which were determined in a similar manner to the inhibition distances. We then used ZT [ 37] to conduct Mantel tests to examine the correlation between matrices of: (1) cultivar genetic distances and cultivar inhibition distances, (2) *Escovopsis* genetic distances and *Escovopsis* inhibition distances, (3) cultivar genetic distances and cultivar attraction distances, and (4) *Escovopsis* genetic distances and *Escovopsis* attraction distances.

## Supporting Information

Figure S1 No-Choice Bioassay II, Interaction and Genetic Matrices Results of no-choice bioassay II (top) were used to generate four interaction matrices (middle). We then used Mantel tests to test for a correlation between interaction matrices and genetic-distance matrices (bottom). Details of the methods can be found in the text.
(1.2 MB EPS)

### Accession Numbers

The GenBank ( http://www.ncbi.nlm.nih.gov) accession numbers for the *Escovopsis* sequences discussed in this study are DQ415661–DQ415670.

## Abbreviations

#days: number of days that it took an *Escovopsis* isolate to reach the end of a given track in a fungal-choice bioassay
AFLP: amplified fragment-length polymorphism
cultivar A: clade-A cultivar
cultivar B: clade-B cultivar
cultivar C: clade-C cultivar
PDA: potato dextrose agar
